# The potential therapeutic effect for melatonin and mesenchymal stem cells on hepatocellular carcinoma

**DOI:** 10.1051/bmdcn/2019090424

**Published:** 2019-11-14

**Authors:** Yasser Mohamed, Mohamed A. Basyony, Nabila I. El-Desouki, Walied S. Abdo, Mohammed A. El-Magd

**Affiliations:** 1 Department of Zoology, Faculty of Science, Tanta University Tanta 31527 Egypt; 2 Department of Pathology, Faculty of Veterinary Medicine, Kafrelsheikh University Kafrelsheikh 33516 Egypt; 3 Department of Anatomy, Faculty of Veterinary Medicine, Kafrelsheikh University Kafrelsheikh 33516 Egypt

**Keywords:** Melatonin, MSCs, HCC, Apoptosis, Inflammation

## Abstract

Background/aim: Herein, we investigated the potential therapeutic effect of Melatonin (Mel) and/or mesenchymal stem cells (MSCs) on rat model of HCC.

Materials and Methods: Female mature rats were divided into 5 groups (*n* = 10/group): normal (Nor), HCC group intraperitoneally injected with 200 mg/kg DEN, and 3 treated groups; HCC + Mel (Mel) group given Mel intraperitoneally 20 mg/kg, twice a week, HCC + MSCs (MSCs) group intravenously injected by 1 × 10^6^ cells, and HCC + MSCs (Mel +MSCs) group.

Results: Rats in HCC group showed most deteriorated effect in form of increased mortality and relative liver weight, elevated serum levels of ALT, AST, ALP, AFP and GGT in addition to increased pre-neoplastic nodules in liver tissues. Liver tissues of HCC group also exhibited lower level of apoptosis as indicated by decreased DNA fragmentation and expression of *p53* caspase 9 and caspase 3 genes and increased PCNA immunoreactivity. Moreover, in this group the expression of *IL6* and *TGFβ1* genes was significantly upregulated. All these deleterious effects induced by DEN were reversed after administration of Mel and/ or MSCs with best improvement for the combined group (MSCs + Mel).

Conclusions: These findings reveal a better therapeutic effect for MSCs when given with Mel and we attribute this beneficial effect, at least in part, to triggering apoptosis and targeting inflammation in HCC. Therefore, combined treatment with Mel and MSCs is recommended to enhance the therapeutic potential against HCC.

## 1. Introduction

Hepatocellular carcinoma (HCC) is a fatal disease that threatens the life of a large sector of the population not only in the developing countries but also throughout the world. It comprises 85-90% of primary liver cancer cases and is the third main cause of cancer death globally. Among the major HCC predisposing factors, infection by hepatitis C and B viruses comes on the top [1]. Failure of HCC treatment is mostly attributed to impairment of liver function, quick development of drug resistance, and the presence of hepatic cancer stem cells [2, 3]. In addition, hepatic cancer cells, similar to other cancer cells, keep their survival through the prevention of apoptosis and maintenance of inflammatory microenvironment [4, 5]. Therefore, to kill cancer cells, these two mechanisms should be specifically targeted. Although most anti-cancer drugs inhibit tumor growth through induction of apoptosis and reduction of inflammation, their uses usually accompanied by severe side effects due to lack of specific targeting [6, 7]. Thus, there is an urgent need to find suitable anti-cancer agents with highest efficacy and lowest adverse effects on normal (healthy) tissues.

Recently, mesenchymal stem cells (MSCs) were successfully used in cell-based therapy for several diseases; however, their potential therapeutic effects are often limited by the inflammatory microenvironment of the hostile tissue [8–11]. Melatonin (Mel), a secretory indoleamine produced from the pineal gland [12] which has antioxidant and anti-inflammatory properties [13], was successfully used to enhance MSCs therapeutic effect against a large variety of diseased conditions [8, 9, 14, 15]. The tumor suppressive effect of Mel was reported against different cancers, and this effect depends mainly on triggering apoptosis and preventing inflammation [16, 17]. Based on the data mentioned above, we hypothesized that co-treatment using MSCs and Mel might improve the therapeutic outcomes (higher apoptosis and suppression of inflammation) against HCC. Therefore, this study was carried out to prove this hypothesis.

## 2. Materials and methods

### 2.1. MSCs culture and characterization

Long bones of male rats were flushed with PBS to obtain MSCs as previously described in our recent publication [18]. Following flushing, filtration (70 mm filter) and centrifugation (2500 rpm for 7 min), the obtained cells were cultured in complete culture media (DMEM, 10% fetal bovine serum and 1% penicillin/streptomycin, Sigma Aldrich), incubated at 37°C and 5% CO2 incubator for 2-3 weeks with frequent changes of media to get rid of non-adherent cells. Adherent cells (at 80-90% confluence) were then harvested by trypsinization and the obtained MSCs were utilized after the third passage. Successful isolation for MSCs was confirmed by flow cytometry (Attune, Applied Biosystem) through detection of CD105 (1:100 dilution, Becton, Dickinson) and CD90 (1:200 dilution, Becton, Dickinson) positive markers for MSCs and hematopoietic stem cells were excluded *via* very low detection of CD45 (1:100 dilution, Becton, Dickinson) using a protocol as previously described [18].

### 2.2. Experimental design

Our experimental protocol was accepted by the Animal Ethics Committee of Kafrelsheikh University. A total number of 50 healthy adult female rats with matched weights (140 ± 5.25) and ages (6 ± 0.12) weeks were housed in plastic cages (25-27˚C and a 12 h light/dark cycle), fed a standard diet ad libitum with free access to water.

The rats were distributed into 5 groups (*n* = 10/group) as follow:

- Normal group (Nor): rats were orally administered saline throughout the experiment (20 weeks).
- HCC group (HCC): rats were intraperitoneally injected once with diethylnitrosamine (DEN; 200 mg/kg in 1 *ml* of PBS, Sigma-Aldrich) and 1 week later, they were orally administrated 2-acetylaminofluorene (2-AAF; 150 mg/kg, Sigma Aldrich) for 2 weeks [19].
- HCC+ Mel group (Mel): HCC rats were intraperitoneally injected by Mel (20 mg/kg, Sigma Aldrich) two times per week from the 9^th^ to 14^th^ week [18].
- HCC + MSCs group (MSCs): HCC rats were intravenously injected by a single dose MSCs (1 × 10^6^ cells/1 ml PBS) at the 12^th^ w [18].
- HCC + MSCs preconditioned with Mel group (Mel + MSCs): MSCs were preincubated with 5 μM Mel for 24 h and then injected as previously mentioned in MSCs group.

### 2.3. Samples collection and preparation

Blood samples and serum preparation were done as previously described [20]. Following sacrificing, the abdomen was incised and the liver was weighed and then thoroughly washed by saline. The liver was divided into two parts, the first part was quickly frozen in liquid nitrogen for RNA extraction and the second was preserved in 10% formalin for histological analysis.

### 2.4. Biochemical analysis

The serum levels of aspartate aminotransferase (AST), alanine aminotransferase (ALT), alkaline phosphatase (ALP), acid phosphatase (AP), α-fetoprotein (AFP), and γ-glutamyl transferase (GGT) were determined using commercial available kits and as previously described [21].

### 2.5. Detection of DNA damage by comet assay

The comet assay was performed on liver tissue as previously described [22, 23]. The migration pattern of DNA fragments of 100 cells was evaluated with fluorescence microscope. The DNA damage index ranged from 0 to 400, where 0 means undamaged DNA with tail length equals to 0. However, 400 refers to highest DNA damage with tail length equals to 4.

### 2.6. Histological and immunohistochemistry analysis

Liver tissue samples were dehydrated in ethanol, cleared in xylene, impeded in paraffin to form tissue blocks, which then sectioned (4-5 μm), finally the slides were stained by hematoxylin and eosin (H & E). Immunostaining was performed as previously described [18, 24] using polyclonal rabbit anti-rat PCNA antibodies (1:500 dilution, Thermo-Scientific, USA) and goat anti-rabbit secondary antibody (1:1000 dilution, Dako, USA).

### 2.7. Molecular analysis by qPCR

Real time PCR (qPCR) was used to detect the altered expression of some genes in liver tissue. We first extracted total RNA from hepaticr tissue using a Gene JET RNA Purification Kit (Thermo Scientific, #K0731, USA) following manufacturer’s protocol and as previously described [25]. The concentration and purity of the isolated total RNA was checked by a Nanodrop (Quawell, Q3000) as previously described [26]. Next, totalRNA was reverse transcribed to cDNA using RevertAid H Minus Reverse Transcriptase (Thermo Scientific, #EP0451, USA). Specific primers for candidate genes (Table 1) were designed by the Primer 3 web-based tool based on the published rat sequence. Finally, qPCR was conducted using, cDNA, primers, and QuantiTect SYBR Green qPCR Master Mix with reaction cycles as previously described [7]. Calculation of relative expression was done using 2^−∆∆Ct^ equation as previously described [27].

**Table 1:** Primers used for qPCR.

Gene	Forward primer	Reverse primer
*p53*	GTTCCGAGAGCTGAATGAGG	TTTTATGGCGGGACGTAGAC
*Caspase9*	AGCCAGATGCTGTCCCATAC	CAGGAGACAAAACCTGGGAA
*Caspase3*	GGTATTGAGACAGACAGTGG	CATGGGATCTGTTTCTTTGC
*IL6*	TCCTACCCCAACTTCCAATGCTC	TTGGATGGTCTTGGTCCTTAGCC
*TGFβ1*	AAGAAGTCACCCGCGTGCTA	TGTGTGATGTCTTTGGTTTTGTCA
*β-actin*	AAGTCCCTCACCCTCCCAAAAG	AAGCAATGCTGTCACCTTCCC

AST serum level (U/L)

ALP serum level (U/L)

### 2.8. Statistical analysis

The statistical analysis was done using one way ANOVA using GraphPad Prism 8 (GraphPad Software, Inc., LaJolla, CA, USA) followed by Tukey’s Honestly Significant Difference (Tukey’s HSD) test. Significance was declared at *P* < 0.05.

## 3. Results and discussion

The isolated MSCs were examined using phase contrast microscope, which showed cells have an identical similarity with MSCs morphological features, including adhesiveness and fusiform shape. This was further confirmed by flow cytometric analysis which showed abundant expression of positive MSC markers CD105 and CD90 and lacked expression of the negative marker CD45 (data not shown).

**Fig. 1:**
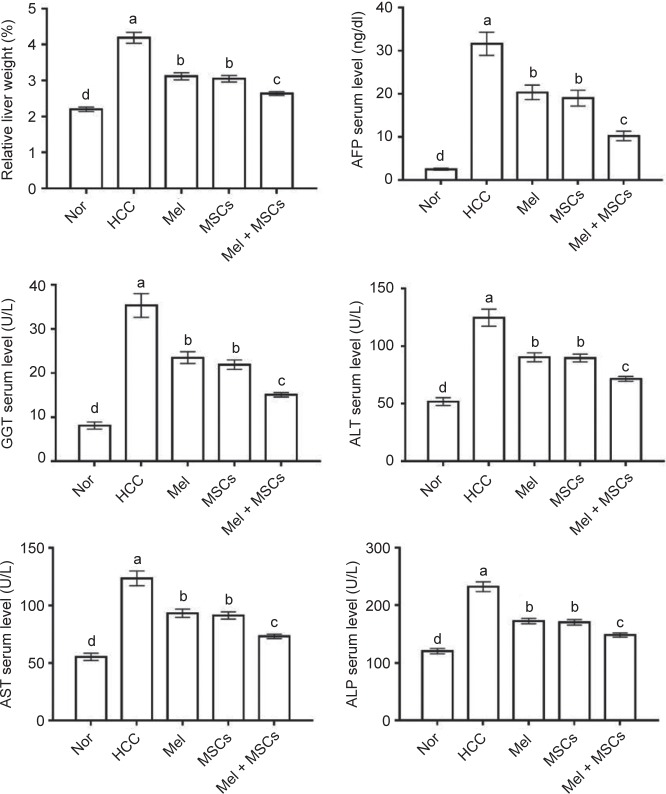
Effect of Mel and/or MSCs on relative liver weight and serum levels of liver cancer markers and liver enzymes (AST, ALT, ALP). Normal control (Nor), HCC, HCC treated by Mel (Mel), HCC treated by MSCs (MSCs), and HCC treated by Mel and MSCs (Mel + MSCs) rats. Values are expressed as mean ± SEM (*n* = 7). Values carrying different lower case letter are significantly different at *P* <0.05.

HCC progression was monitored by mortality rate, change in liver relative weight and serum levels of liver cancer markers (AFP and GGT). No mortality was recorded in all groups except HCC group, which showed a mortality rate of 20% (2/10). Expectedly, HCC group exhibited the highest relative liver weight when compared to the other groups (Fig. 1). In contrast, HCC rats treated with Mel and/or MSCs showed significant lower relative liver weight with lowest weight in Mel pretreated MSCs group. As changes in mortality rate and relative liver weight are not enough to guarantee low carcinogenicity, we further estimated the serum levels of AFP and GGT and notably found an association between this increase and AFP and GGT elevated serum levels in the HCC group. Again, treatment with Mel and/or MSC resulted in a significant decrease in AFP and GGT, with lowest levels in combined group (Fig. 1).

The effect of Mel and/or MSCs treatment on HCC was monitored by detection of fluctuations in serum levels of ALT, AST, and ALP. As expected, HCC group exhibited higher serum levels of these enzymes, indicating liver dysfunction [28]. However, treatment with Mel and/or MSC decreased this elevated levels to levels comparable to that of normal control group, with best effect to the combined treated group (Fig. 1). Collectively, these findings indicate that cotreatment of MSCs with Mel gives better therapeutic effect against HCC than each alone. Similar results were reported in rat model of HCC induced by CCL4 [29], liver injury [30], and chronic liver disease [31].

**Fig. 2:**
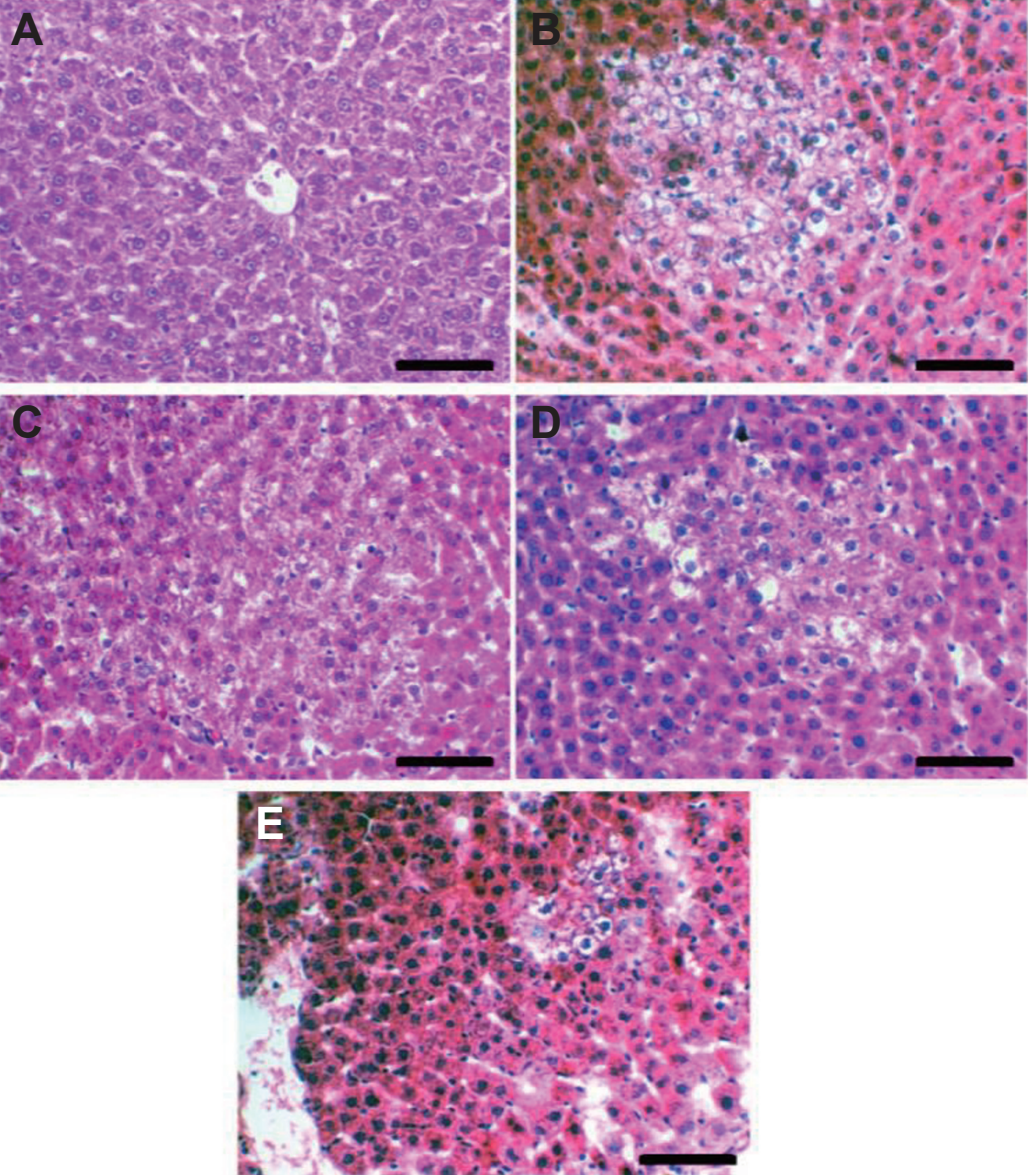
Photomicrographs of liver sections stained by H & E in normal (A), HCC (B), Mel (C), MSCs (D), and Mel + MSCS (E) groups. Scale bar = 50 μm.

**Fig. 3:**
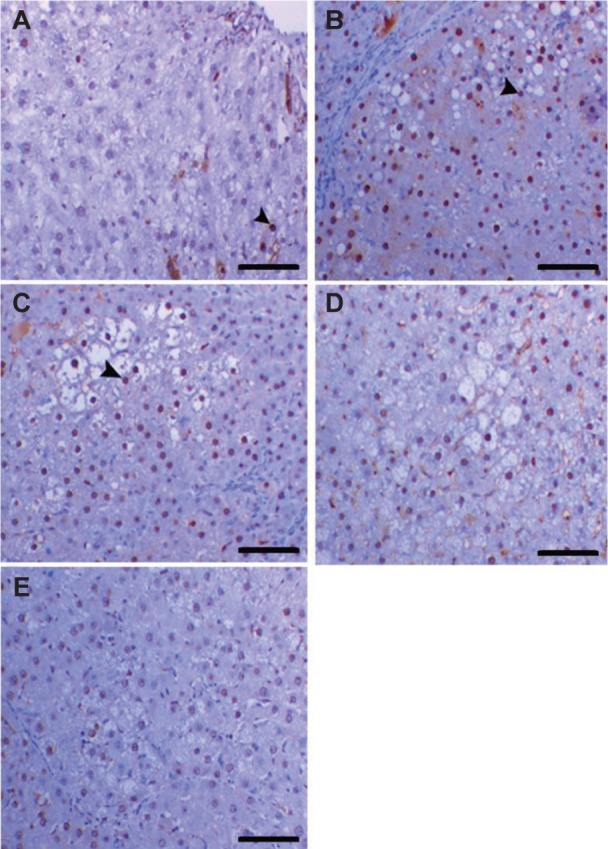
Photomicrographs of liver sections immuostained with PCNA antibody in normal (A), HCC (B), Mel (C), MSCs (D), and Mel + MSCS (E) groups. Scale bar = 50 μm.

Unlike in normal group (Fig. 2A), rat liver in HCC group had pre-neoplastic nodules with altered hepatic foci (AHF) that characterized by presence of a large number of hexagonal, vacuolated cells and oval cells (Fig. 2B). In contrast, treatment with Mel and/or MSCs deceased these AHF and increased apoptosis especially in combined treated group (Fig. 2C-E). In agreement, Mel has been shown to decrease DEN-induced HCC [32], and ovarian cancer [33] in rats. Similar AHF were also observed in HCC rat model induced by other carcinogens [34]. These distorted liver histological architecture observed in HCC group plays an integral role in elevation of liver enzymes in blood. Damage of tissues, especially cell membrane, lead to release of these enzymes from the cells to circulation [35]. A very low PCNA immunostaining reactivity was noticed in liver of the control group (Fig. 3A). However, HCC rats exhibited extensive PCNA immunostaining reactivity (Fig. 3B). Rats treated with Mel and/or MSCs showed a decrease in PCNA labelled cells, with a notable reduction in DEN and Mel group, relative to HCC group (Fig. 3C-E).

**Fig. 4:**
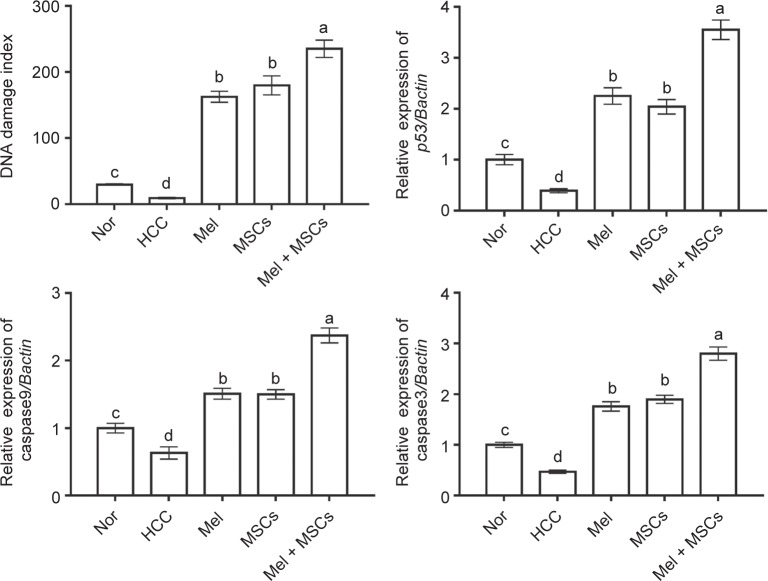
Effect of Mel and/or MSCs on DNA fragmentation as determined by comet assay and on expression of apoptotic genes, p53, caspase 9 and caspase 3, in livers of HCC rats as detected by qPCR. Data presented as fold change from the normal (Nor) control group. Values are expressed as mean ± SEM (*n* = 7). Values carrying different lower case letters are significantly different at *P* < 0.05.

Previous studies have reported anti-cancer effect for Mel, at least in part, through induction of apoptosis in cancer cells [16, 17]. In this study, we evaluated this apoptotic effect by assessing DNA fragmentation (by comet assay), and changes in expression of apoptotic genes, p53, caspase 3 and caspase 9, (by qPCR). DNA damage was significantly increased after administration of Mel and MSCs alone or in combination, as revealed by a high damage index compared with that in the HCC group (Fig. 4). Again, the highest DNA damage was noticed in animals given both Mel and MSCs. However, no significant difference in DNA damage was noticed between the Mel group and the MSCs group. In agreement, administration of Mel induced higher DNA fragmentation (higher TUNEL-positive cells) in ovarian cancer [33]. It also inhibits progression of HCC both *in vitro* [16] and *in vivo* [32], *via* apoptosis.

Livers of HCC rats exhibited a significant downregulation in the proapoptotic genes *p53*, caspase 3 and caspase 9 relative to normal control rats (Fig. 4). This reduced expression was significantly increased following administration of Mel and MSCs each alone or combined, with best results in the combined group. This infers Mel and MSCs ability to trigger apoptosis in liver of DEN-treated rats, with better effect in the combined group. In consistent with our results, Mel was reported to induce expression of p53, and caspases genes and proteins in several cancer cell lines and livers of HCC rats [16, 32]. In contrast to its apoptotic effect on cancer cells, Mel has anti-apoptotic effect on healthy cells and induces their proliferation [36], suggesting influence on cell viability according to the type of target cells.

**Fig. 5:**
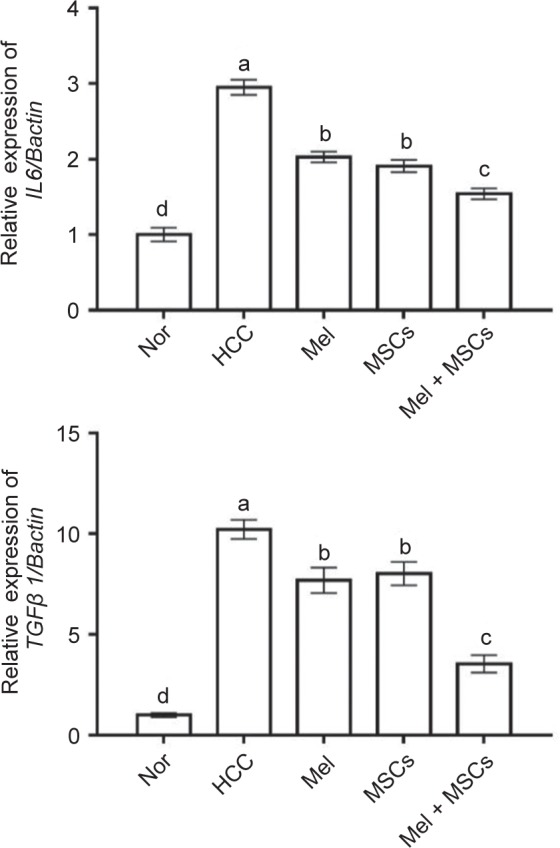
Effect of Mel and/or MSCs on expression of inflammation-related genes, IL6, TGFβ1, in livers of HCC rats as detected by qPCR. Data presented as fold change from the normal (Nor) control group. Values are expressed as mean ± SEM (*n* = 7). Values carrying different lower case letters are significantly different at *P* < 0.05.

Another possible way by which cancer cells maintain their high proliferative capacity and survival, is induction of inflammation [37]. Therefore, inhibition of inflammation is an urgent need for killing cancer cells. Melatonin can relieve liver diseases through inhibition of inflammation [30]. To check whether Mel and/or MSCs have anti-inflammatory effect on HCC, expression of the inflammation-related genes (*IL6* and *TGFβ1*) was determined. Rats with HCC exhibited the highest expression of IL6 and *TGFβ1* as compared to other groups. In contrast, administration of Mel and MSCs alone or together significantly decreased this elevated expression, with better effect in the combined group (Fig. 5). In consistence, Mortezaee, *et al.* [19] also showed downregulated expression of *TGFβ1* gene in liver of CCL4-induced HCC rats following treatment by MSCs pretreated by Mel. *TGFβ1* is the most important cytokine pathogenesis of liver fibrosis which precedes HCC [32]. The important question is why the best effect was noticed when MSCs cotreated with Mel before administration. It is possible that Mel can directly activate MSCs proliferation. In support to this notion, previous studies have shown that Mel can bind to its receptors in MSCs and subsequently triggers cell viability [38, 39]. In addition, Mel can regulate stem cell proliferation [38, 39], motility [40], and differentiation into hepatocyte-like cells [31]. Another possible mechanism is the ability of Mel to improve the hostile hepatic tissue for MSCs homing and proliferation. The hostile hepatic microenvironment in HCC (high inflammation and hypoxia) induces apoptosis in MSCs thereby, restricting their therapeutic potential [41]. Mel is well known for its anti-inflammatory effect and so when given with MSCs, it will decrease the inflammation and so maintain MSCs viability. For this reason, most of recent studies used combined Mel and MSCs therapy, for example in the treatment of liver fibrosis [29, 31] and heart ischemia [14]. Application of this treatment strategy may also need fewer MSCs for homing, and so this will decrease MSCs accumulation in other organs.

The results obtained from our study and those by previous studies revealed that a single dose of MSCs can improve HCC [5]. However, treatment using a single dose of MSCs is doubtful as this concentration could not be enough to obtain a sustained effect. Until now, no available information on the *in vivo* influence of different doses of MSCs on HCC. Hence, further studies are needed to unveil an appropriate treatment regimen for MSCs to sufficiently indicate number and interval of doses and route of administration.

## 4. Conclusions

The present study reported that co-treatment of MSCs with Mel enhances the anti-HCC effect of MSCs *via* triggering apoptosis and preventing inflammation. The results of this study may be valuable in treatments targeting liver diseases in patients. However, further molecular investigations (especially on microRNAs, and long non coding RNAs) are required to give more details on how Mel pretreatment maximizes the therapeutic potential of MSCs.
